# Epidemiology of Chronic Effects of Traumatic Brain Injury

**DOI:** 10.1089/neu.2021.0062

**Published:** 2021-08-17

**Authors:** Juliet Haarbauer-Krupa, Mary Jo Pugh, Eric M Prager, Nicole Harmon, Jessica Wolfe, Kristine Yaffe

**Affiliations:** 1 Division of Injury Prevention, National Center for Injury Prevention and Control, Centers for Disease Control and Prevention, Atlanta, Georgia, USA.; 2 Informatics, Decision-Enhancement and Analytic Sciences Center, VA Salt Lake City, Salt Lake City, Utah, USA.; 3 Department of Internal Medicine, Division of Epidemiology, University of Utah School of Medicine, Salt Lake City, Utah, USA.; 4 Cohen Veterans Bioscience, New York, New York, USA.; 5 Department of Neurology, University of California San Francisco, San Francisco, California, USA.; 6 San Francisco Veterans Affairs Medical Center, San Francisco, California, USA.; 7 Departments of Epidemiology/Biostatistics and Psychiatry, University of California San Francisco, San Francisco, California, USA.

**Keywords:** clinical trial designs, epidemiology, patient selection criteria, traumatic brain injury

## Abstract

Although many patients diagnosed with traumatic brain injury (TBI), particularly mild TBI, recover from their symptoms within a few weeks, a small but meaningful subset experience symptoms that persist for months or years after injury and significantly impact quality of life for the person and their family. Factors associated with an increased likelihood of negative TBI outcomes include not only characteristics of the injury and injury mechanism, but also the person’s age, pre-injury status, comorbid conditions, environment, and propensity for resilience. In this article, as part of the Brain Trauma Blueprint: TBI State of the Science framework, we examine the epidemiology of long-term outcomes of TBI, including incidence, prevalence, and risk factors. We identify the need for increased longitudinal, global, standardized, and validated assessments on incidence, recovery, and treatments, as well as standardized assessments of the influence of genetics, race, ethnicity, sex, and environment on TBI outcomes. By identifying how epidemiological factors contribute to TBI outcomes in different groups of persons and potentially impact differential disease progression, we can guide investigators and clinicians toward more-precise patient diagnosis, along with tailored management, and improve clinical trial designs, data evaluation, and patient selection criteria.

## Introduction

Traumatic brain injury (TBI) is a global public health concern and one of the leading causes of death and disability, with an estimated 64–74 million persons sustaining a TBI each year.^1^ TBI is traditionally identified as mild, moderate, or severe at the time of injury by a measure called the Glasgow Coma Scale (GCS). Although most of the literature on the outcomes and long-term effects of TBI focus on moderate-to-severe cases, mild TBI (mTBI) has the highest rate of emergency department (ED) visits. Notably, “mTBI,” “concussion,” and “minor head injury” are often used interchangeably, but in this report we use mTBI.^2–6^ Internationally, the majority of TBIs (75–90% of reported cases as assessed by the GCS) tend to be mild.^7,8^ Globally, total cases are likely underestimated, given that many mTBIs go undetected, untreated, and unrecorded because of the lack of a national TBI registry that could be used to obtain comprehensive data on TBI. Although the lifetime prevalence of mTBI is difficult to assess, a global incidence rate is estimated at 100–749 cases per 100,000 persons,^9–14^ or ~55.9 million persons.^1^ In the United States, an estimated 2.87 million TBI-related ED visits (~2.5 million TBI ED visits with ~1% being for mTBI, 288,000 TBI-related hospitalizations, and 56,000 TBI-related deaths) were reported in 2014^15^; ~43% of these will experience long-term disabilities.^16,17^

TBIs are attributed to several mechanisms of injury, including falls, motor vehicle or other types of road injuries, sports-related injuries, and interpersonal physical violence or violence by other means (e.g., blast injury). These injury mechanisms vary by geographical region, socioeconomic factors, age, and sex. Indeed, stratifying by geographical region can illuminate clear differences in injury type and overall burden of disease.^18^ For example, incidence of TBI—of all severities—is highest in the United States and Canada, but Southeast Asian and Western Pacific regions experience the greatest overall burden of disease.^1^ Low- and middle-income countries experience nearly 3 times more TBI cases proportionally than high-income countries; this is attributed to a higher prevalence of risk factors for TBI causes (e.g., motor vehicle crashes) and differences in health systems for patients to seek medical care and address associated health effects. With respect to age, TBI shows a bimodal distribution with the highest incidence in the youngest and oldest age groups.^15^ These age groups are likely more susceptible to TBI because of increased risk for various causes of injury, such as falls in children under the age of 4 (839 per 100,000) and falls in the elderly over the age of 75 (599 per 100,000), as well as motor vehicle crashes in adults ages 15–24 (236 per 100,000).^15^

Mild TBI makes up 10–15% of all sports-related injuries across the age span^19^; ~283,000 children seek care each year at an ED because of sports-related TBI, with the highest rates among males and children between 10 and 17 years of age.^20^ Although injuries from non-sports activities occur across children’s life spans, sports-related injuries increase around age 6 and peak during the teenage years, a time when children are engaging more frequently in organized sports.^21^ Males account for a significantly greater proportion of all TBIs than females, with annual incidence rates being 388 per 100,000 males versus 195 per 100,000 females at all ages.^12,22^ Whereas males have a higher incidence of concussion in some contact sports such as football,^20^ females have a higher rate in sex-comparable sports such as soccer.^23^

Incidence of TBI is higher in the military compared to the civilian population.^24^ This is attributable, in part, to the physically demanding and potentially dangerous operational and training activities and the associated risk for blast exposures from improvised explosive devices, suicide bombers, land mines, mortar rounds, and rocket-propelled grenades.^25^ Incidence is higher for those in the Army compared to the Navy, Marines, or Air Force and more so for those on active duty than those who are not on active duty. From 2000 through the third quarter of 2019, >413,000 U.S. military personnel worldwide were diagnosed with a TBI; ~83% of cases were considered mild, 10% moderate, and 1% penetrating or severe.^26^ Similar to civilian populations, females in the military experience TBI at a lower rate, but report more neurobehavioral symptoms after mTBI than their male counterparts,^27–33^ likely, in part, because they often experience TBI secondary to interpersonal violence or military sexual trauma, but also because they are more likely to be screened for TBI than males.^34,35^

Despite the many epidemiological studies focused on measuring the prevalence of TBI, the true prevalence of TBI is still unknown among populations for which access to care may be limited (e.g., underserved or rural populations) or for which reporting of TBI may be viewed as detrimental to career aspirations (e.g., military, athletics). In addition, the statistics/incidence rates described above indicate the acute effects of exposure to a TBI; 80–85% of those who sustain an mTBI fully recover within 3 months.^36^ What remains less understood is the incidence, prevalence, and risk factors for the chronic effects of TBI. In the following sections, we summarize the current literature regarding the epidemiology of chronic TBI within key populations and describe how the long-term sequelae of TBI both present with overlapping symptoms and are associated with increased comorbidity with other diseases. We then provide considerations for designing studies that more fully account for the long-term consequences of TBI.

## Variables That Impact Traumatic Brain Injury and Outcomes

TBI is increasingly described as a chronic health condition, given that evidence indicates that the health effects of TBI can persist over time, particularly for persons who experience moderate-to-severe TBI.^37,38^ Many persons with mTBI recover within a short time frame, but recent studies indicate that the effects of a mTBI in children and adults can persist for a year or longer.^39–42^ These findings indicate the need for improved follow-up and management pertinent to the age of the patient.^43,44^

Although discussing all of the factors that affect TBI recovery is beyond the scope of this article, we highlight a few critical sociodemographic categories that are especially meaningful across the life span. Injury-related, personal, and environmental variables can have a strong impact on an individual course of recovery in children.^45^ Indeed, reports have shown that pre-injury functioning, family factors such as parenting styles, and intellectual ability can predict outcome,^46,47^ highlighting the importance of these variables in reducing the impact of a pediatric TBI. For example, one study explored health inequities experienced by children with chronic health conditions, including TBIs of all severities, and how the inequities affect long-term mortality and chronic symptoms.^48^ The researchers identified serious racial and ethnic health and healthcare inequities in these children. In addition, a 2006 review noted that black children experience more TBI-related hospitalizations and have a higher TBI-related mortality rate than children who are white.^49^ These findings suggest that general health disparities experienced by minority children may increase the likelihood that those with a TBI will experience condition-related health disparities.

Consistent with this concept, children who have private health insurance show lower mortality rates after TBI than those with public insurance or those who are un-insured; further, there is a higher TBI prevalence in states with greater levels of private insurance, suggesting inadequate recognition of TBI among children with less access to care.^50^ Disparities also exist across the spectrum of care for adults with TBI, partially attributable to the heterogeneity of available resources. In rural versus urban settings in the United States, TBI (in most cases severe TBI, though it is not clearly reported) is associated with higher mortality rates.^51^ This could be a product of reduced access to pre-hospital care, trauma centers, neurosurgical interventions, or rehabilitative services. However, people with a TBI who live in rural areas report equivalent satisfaction with their quality of life despite having potentially lower levels of medical resources and reduced social participation.^52^

Recent studies on adults with mTBI seeking care in EDs revealed several issues related to their care and outcomes: 1) pre-injury factors such as mental health diagnosis, medical history of other health conditions and comorbidities, and previous TBIs are associated with persistent symptoms after mTBI^53,54^; 2) lingering functional impairments influence emotional health after mTBI^55^ and can contribute to other injury-related difficulties that affect a patient’s lifestyle at 1 year post-injury^55,56^; and 3) these factors can compound and influence other socioeconomic factors, such as employment and insurance, but also necessitate the need for medical follow-up.^57^

Sex must also be considered when describing the impact and outcomes of TBI. Females comprise approximately half of all TBI-related ED visits, 41% of TBI-related hospitalizations, and 27% of TBI-related deaths.^58,59^ However, these may be underestimates given that a brain injury may go unreported or undetected, especially when the injury is mild. Indeed, although there are no national prevalence estimates for TBI as a result of intimate partner violence, studies in shelters or EDs show that 30–74% of women who experience intimate partner violence have a history of TBI, highlighting the importance and frequency of this occurrence. When women do go to the ED, healthcare providers might not recognize that the symptoms experienced by victims of interpersonal violence could be the result of a TBI.^60^ In addition to specific types of violence contributing to TBI, hormonal factors may impact recovery outcomes in females. Although the research is still in its infancy and pre-clinical and clinical studies are necessary to determine the underlying neuroprotective effects of hormones,^61^ it is clear that circulating estrogens may elicit a differential pain response^62,63^ and the reduction of progesterone concentration after TBI can lead to a withdrawal from its proposed neuroprotective properties.^64^

## Long-Term Sequelae of Traumatic Brain Injury

Patients of all ages often present with a heterogeneous combination of symptoms that can progress in nature and involve multiple pathophysiological mechanisms after a brain injury of varying severity.^65–67^ These biological mechanisms can cause excitotoxicity, apoptosis, inflammatory events, seizures, demyelination, white matter pathology, and neurodegeneration, resulting in prolonged motor and cognitive deficits.^65,68^ Some TBI symptoms may occur early and improve or worsen over time, whereas others may develop later in the course of recovery.^67^ Although symptoms resolve for a majority of persons who experience mTBI in ~3 months, some continue to experience symptoms at or beyond 1 year post-injury.^69^ Estimates indicate that between 15% and 30% of persons who experience an mTBI suffer symptoms long after exposure,^70–74^ as indicated by imaging findings, age, population studied, time of initial assessment, and patient presentation.^75^ Although these percentages are likely an underestimate, studies are beginning to reveal that even a single mTBI might manifest as a measurable cognitive impairment as early as 3 months after injury. Unfortunately, research exploring the long-term consequences of mTBI remains in its infancy. Moreover, disputes in the interpretation of data have arisen because of insufficient methods to detect subtle changes, flaws in quantitative analyses,^75,76^ and failure to account for the heterogeneity in symptom presentation, sociodemographic factors, environmental factors, and treatment/outcome trajectories.

The sections below break down the chronicity of TBI into subpopulations, although each of these groups shows even further heterogeneity in the presentation and outcomes of the disease. Exploring the variations in presentations can facilitate approaches to achieve better follow-up over time and target therapies toward specific patient populations using precision medicine.

## Long-term outcomes in children

Every year, ~500,000 children ≤14 years of age sustain TBIs severe enough to require visits to the ED,^77^ with young children 0–4 years of age having the highest rate of ED visits.^15^ Similar to the general population, symptoms subside in most pediatric patients with mTBI within a couple weeks of injury and between 70% and 80% fully recover within 3 months.^78–80^ Those who have persistent symptoms after 3 months usually report headaches, fatigue, and frustration, with ongoing headaches being the most common symptom.^81^ A number of wide-ranging factors impact the persistence of symptoms, including age, sociodemographic factors (e.g., race/ethnicity), comorbidities with other mental health or neurological disorders, learning difficulties, and family and social stressors.^82^ These factors can lead to long-term detriments in a range of functions, including school performance and social participation,^83^ as well as long-term impairments in health, cognitive, emotional, behavioral, family, and other social outcomes (for a review, see a previous work^45^).

Relatively few studies have examined the long-term consequences of mTBI in children.^15^ However, delayed sequelae from mTBI have been reported in children (either from sports-related events or non-sports-related events such as falls or motor vehicle crashes). Indeed, recent reports indicate persistent or emerging deficits in cognition, behavior, and mental health^39,42^; these deficits appear to be independent of the injury mechanism, duration of time post-injury, and age at the time of injury.^39^ Emerging research has also established that after a mTBI, children (and adults) have difficulty with visual smooth pursuit, saccades, vestibular-ocular reflex, convergence, and gait at the time of diagnosis.^84,85^ These vestibular and oculomotor impairments may be associated with worse outcomes and, given that they can be identified early in the clinical profile, may help to better inform treatments and interventions early after an injury.^84,86^

Some deficits may persist or appear later in time as a result of developmental processes involved in brain maturation and might interfere with academic achievement, disrupt psychosocial functioning regardless of injury severity,^42,45,87,88^ or increase susceptibility to a variety of health and neuropsychiatric conditions, including learning disorders (21%), attention-deficit/hyperactivity disorder (ADHD; 21%), speech/language problems (19%), developmental delay (15%), bone, joint, or muscle problems (14%), and anxiety problems (13%).^50^ Importantly, these findings indicate that comorbid conditions might occur at higher rates; indeed, one study found that when a child under the age of 10 (and especially if the injury occurred before 5 years of age) experienced a mTBI sufficient to warrant temporary hospitalization, they were likely to show adverse psychosocial outcomes (e.g., hyperactivity/inattention) and present with conduct disorder behaviors at ages 10–13.^42^ In addition to longer-term effects, children who experience an injury across the pediatric life span, including those in elementary school, are at higher risk for exposure to additional mTBIs, which can impact their outcomes.^85,89^

A few studies have summarized the chronic features observed in pediatric mTBI (see an earlier work^45^ for a review), and a limited number of studies have assessed outcomes in adults who experienced a TBI as a child. For example, a recent study examined adults who experienced a childhood TBI in a Swedish birth cohort, comparing them to siblings who did not experience an injury.^90^ Findings from this and similarly designed studies indicate that persons who experience a childhood TBI have lower rates of enrollment in post-secondary education, are less likely to be employed or live independently, and are more likely to work in entry-level or low-skilled jobs when compared to those with other disabilities or persons who have not experienced a TBI.^19,90–96^

Biological and environmental circumstances are difficult to separate as risk factors for TBI outcomes. Studies have reported a high prevalence of TBI in young persons entering the justice and prison systems. For example, a 2015 study evaluated the prevalence of TBI based on self-reported history among 93 male participants 15–18 years of age in a juvenile facility. Approximately 82% had experienced at least one TBI that resulted in being knocked out or dazed and confused, and 44% reported ongoing symptoms.^97^ Longitudinal studies have found associations between TBI in childhood and subsequent increased criminality and conduct problems later in life.^98^ Moreover, a cohort study of prison inmates found that those with a self-reported TBI history were more likely to be rearrested sooner than those without a TBI history.^99^ This association between TBI and criminality could be the result of long-term TBI-related impairments that affect the person’s ability to regulate behavior and affect attention capacity and/or interpersonal skills. Alternatively, the TBI might have been the result of novelty seeking and low harm avoidance in persons prone to risky behaviors. Indeed, a larger proportion of juvenile offenders who have experienced a TBI were injured because of a fight, road traffic accident, or fall compared to non-offenders.^97^ However, history of substance use is also associated with TBI and juvenile delinquency, making these relationships more complex.^100^

Although the majority of pediatric mTBI studies report on psychosocial outcomes, many gaps remain in this research. Indeed, no study to date has assessed: 1) longitudinal changes in the developing brain after mTBI; 2) age-related differences in injury mechanisms contributing to long-term consequences; 3) long-term anatomical changes associated with injury (e.g., diffuse axonal injury, swelling); and 4) genetic and sociodemographic factors contributing to long-term consequences. Further, two studies have noted that the existing research on mTBI in children suffers from severe methodological shortcomings, including: 1) variable definitions of mTBI; 2) lack of inclusion/exclusion criteria; 3) the underlying assumption that children with mTBI are a homogenous group; 4) lack of inclusion of a control group with no TBI or other injury; 5) failure to control for pre-injury risk factors; 6) inadequate sample sizes; and 7) lack of standardized tests to measure outcomes and longitudinal designs.^75,80^

## Long-term outcomes in adults

As previously discussed, men are more likely at every age to experience TBI than women, although women are more likely to report chronic sequelae; less-educated persons and those with a previous mental health diagnosis are also more likely to report persistent symptoms.^22,101^ Symptoms commonly associated with chronic sequelae include cognitive (e.g., attention difficulties, memory problems, and executive dysfunction) and social deficits.^102^ Indeed, an age-matched prospective cohort study assessed long-term social cognition after mTBI in adult civilians.^103^ Four years after injury, the TBI group showed a significant reduction in social inference—interpreting verbal and non-verbal social cues—even after controlling for cognitive functioning, suggesting that even mTBI can potentially impact overall functioning. In another study using mild-to-moderately injured patients, younger adults (ages 23–63 years) reported more psychological symptoms, such as anxiety, whereas older adults (65–91 years of age) reported more physical symptoms, such as fatigue, balance, and coordination problems.^44^ Other studies have found that mTBI across the age span is associated with persistent headache,^104^ vestibular dysfunction,^105^ depression, and cognitive complaints.^106^ Adults with sports-related concussion compared to those without a concussion history showed significant cognitive deficits in verbal memory, recall, and attention 10 years after retirement.^107^

Studies assessing the long-term (>1 year) consequences of mTBI in adults are sparse. The paucity of longitudinal research is complicated by 1) lack of a clear definition of post-TBI brain symptoms or interpretation of individual reports; 2) lack of standard controls (many controlled studies use injured patients spared of TBI); 3) selection bias; and 4) poor follow-up. Taking many of these factors into consideration, studies have revealed that patients who have three or more symptoms at the time of injury also report new or worse symptoms 1 year post-injury.^108,109^ More recently, investigators from the Transforming Research and Clinical Knowledge in TBI (TRACK-TBI) study found that less than half of adults with mTBI who present to a level 1 trauma center return to pre-injury levels of daily functioning at 1 year based on self-report.^56^ The factors contributing to delayed or incomplete recovery include comorbid psychiatric disorders, age at time of injury (see more below), abnormal acute neuroimaging, expectation of poor outcomes, and repetitive injuries (see an earlier work^110^ for additional details).

It is beyond the scope of this article to explain how each factor contributes to delayed or incomplete recovery; however, it is critical to highlight two factors reported in the literature: repetitive injury and age. Although the prevalence of repetitive mTBI is less clear, it is understood that previous TBI is a risk factor for future TBI and incomplete recovery.^111^ Indeed, multiple insults can produce cumulative effects and lead to long-term consequences, including age-related neurodegenerative disorders.^112^ Repetitive injuries, including hits to the body that can cause subconcussive TBIs, occur mostly through contact sports and do not produce gross structural or detectable functional changes in the brain parenchyma. However, the cumulative effects may result in four major clusters of symptoms: cognitive, behavioral, mood, and motor disturbances.^13^ Repetitive injury has been assessed most thoroughly among contact sports such as American football. These athletes have increased risk for death by suicide, diminished cognitive functioning, macrostructural, microstructural, functional, and neurochemical changes, and increased propensity for death from neurodegenerative causes such as dementia or Alzheimer’s disease.^19,113,114^

Older persons are more likely to sustain a TBI by falling,^115^ be hospitalized following their injury,^67,115^ and suffer from severe injuries as a consequence of their fall.^44,111^ Given that cognitive and physical abilities in adults diminish with age, the lasting consequences of a TBI of any severity might result in a greater impact on daily living. Among persons who receive inpatient care for a moderate-to-severe TBI, older persons are more likely to die within 5 years.^67^ In addition, older age groups show a greater amount of decline in cognitive and motor outcomes after a TBI compared to their younger counterparts with similar-severity injuries and require more assistance or supervision with increasing age >50 years. In a similar cohort, older persons were associated with poorer medical, functional, and participation outcomes than their younger counterparts.^116^ These more-severe outcomes suggest that older persons are more susceptible to experiencing a TBI, have a higher incidence of ED visits for TBI, are more vulnerable to the deleterious effects of TBI, and/or have symptoms that are exacerbated by the state of their health going into the TBI.

## Long-term outcomes in military populations

As in civilian populations, the majority of military-related TBIs are classified as mild.^117,118^ Unfortunately, the long-term clinical impact of military-related injuries remains incompletely described, most likely because the studies are restricted to single-cohort evaluations, retrospective analyses, and self-reports.^119,120^ Moreover, similar to civilian samples, there are few standardized requirements for diagnosis or available treatments for TBI, leaving many injuries unreported. Despite the failures of previous studies, research assessing TBI in the military population have consistently reported poorer outcomes on the Glasgow Outcome Scale-Extended compared to civilian studies at all severities.^120–124^ One prospective study of active-duty U.S. military personnel examined longitudinal outcomes among four groups of combat-deployed service members: control (no TBI or blast), blast control (no TBI), mTBI only, and blast plus mTBI. The analyses found that service members with mTBI and those with blast plus mTBI had significant neurobehavioral impairment and more-severe depression and post-traumatic stress disorder (PTSD) symptoms compared to combat-deployed controls; outcomes for blast controls fell between those two groups and controls.^120^

Another longitudinal study compared 5-year clinical outcomes of active-duty U.S. military after mTBI and blast injury to combat-deployed control persons.^125^ Participants in the mTBI group had no previous history of TBI. The study found that global disability, satisfaction with life, neurobehavioral symptom severity, psychiatric symptom severity, and sleep impairment were significantly worse in those with blast mTBI compared with combat-deployed controls. These differences in outcomes suggest that the underlying mechanisms and etiology may differ based on the injury type and that treatment options that work in civilians may not necessarily translate to the military population.

Some aspects of the chronic impact of combat-related TBI are focused on social function. Although the majority of these studies use moderate-to-severe TBI cohorts, the trends appear to be similar even with mild injuries.^126–128^ Community reintegration studies suggest differences in social function between civilian and military populations with TBI. Indeed, approximately half of veterans with TBI report difficulty readjusting to civilian life, with even worse social function among those who are also diagnosed with PTSD.^129,130^ Civilian studies have found that TBI itself, rather than mental health, is associated with less-favorable community reintegration outcomes.^126–128,131^ Similarly, a study in veterans confirmed that military TBI exposure is associated with long-term impacts on social and family functioning, community reintegration, and the ability to return to work, even after controlling for PTSD comorbidity and combat experiences.^129^ Sociodemographic characteristics may also account for some of these outcomes. For example, veterans with lower education report lower levels of social support and family functioning, and women report significantly more difficulty with community reintegration compared to men.^129^

## Comorbidities with Traumatic Brain Injury

A significant long-term consequence of TBI is the association with and susceptibility to comorbid psychiatric and neurological conditions. As described in previous sections, several health conditions are associated with risk for TBI and outcomes after TBI. These complex patterns of comorbidity complicate diagnosis and treatment and suggest that the disease course may be modifiable. Given that the field is moving toward an era of precision medicine, this raises the question: How can researchers assimilate this complexity to appropriately evaluate individual presentations and, ultimately, better treat patients and improve health outcomes?

## Association of traumatic brain injury with dementia

TBI in adults has been identified as an early risk factor for Alzheimer’s disease and dementia.^132^ This phenomenon has been well documented in veterans who sustained a TBI of any severity.^112,133–138^ Unfortunately, findings are mixed and can depend on the study design. For example, two prospective studies failed to find an association between TBI and Alzheimer’s disease.^139,140^ though the former indicated that TBI with loss of consciousness (LOC) is associated with risk for Lewy body accumulation, parkinsonism, and Parkinson’s disease, but not non-parkinsonism dementia, Alzheimer’s disease, neuritic plaques, or neurofibrillary tangles.^139^ By contrast, multiple retrospective studies have found significant correlations between mTBI and increased risk for dementia. Indeed, a retrospective cohort study of 188,764 U.S. veterans >55 years of age examined the association between TBI and the risk of all-cause dementia.^136^ mTBI was associated with a 60% increased risk of developing dementia over 9 years, after accounting for competing risks and potential confounders.^136^ Importantly, the results showed an additive association between mTBI and other conditions, such as PTSD and depression, on dementia risk. A subsequent study examined the role of TBI severity in the association between TBI and dementia diagnosis in veterans.^112^ Whereas mTBI without LOC was associated with a >2-fold increase in the risk of dementia diagnosis, consistent with an independent report,^141^ those with moderate or severe TBI had a nearly 4-fold risk of dementia.

Another recent study used national registries to form a very large cohort (>2 million people) and found an association between mTBI and increased risk of dementia and Alzheimer’s disease when compared to persons with non-TBI trauma.^133^ Risk of dementia in older adults was the highest the first 6 months after injury and increased with an increasing number of TBI events. However, age and sex must also be taken into consideration. For example, younger age of sustaining a TBI was associated with greater risk for subsequent dementia, but patients with mTBI only showed an increased risk for dementia at ≥65 years. This finding is in line with the study described above^133^ and other studies that only included persons >65 years of age.^142^ Finally, a cohort study examined whether TBI, PTSD, or depression increase dementia risk among older female veterans.^143^ The results showed that women with these conditions had a 50–80% increased risk of developing dementia compared to women without these diagnoses; those with more than one condition had a 2-fold increased risk of dementia. Research on the association of TBI and dementia is complex, with inconsistencies in study findings and evolving results that can be controversial.

## Association of traumatic brain injury with psychiatric disorders

The long-term neuropsychiatric sequelae of TBI seldom occur in isolation, with between 30% and 50% of adults with a moderate-to-severe TBI presenting with a psychiatric illness, such as depression after trauma.^144^ Evidence suggests that TBI increases the risk of developing mood and anxiety disorders, substance-use disorders, and psychosis.^145^ In a study of 60 persons who suffered from a TBI of any severity, around half developed a new psychiatric disorder after injury, with significantly higher lifetime rates of depression (26% vs. 8%), panic disorder (8% vs. 1.6%), and psychotic disorders (8%) than base rates.^146^ In the Epidemiologic Catchment Area study, severe TBI with LOC or confusion was associated with major depression and anxiety disorders.^147^ TBI is also associated with chronic pain,^148^ suicidality,^149^ substance-use disorders,^150^ and sleep disturbances.^151^ Other studies have indicated varying rates of psychiatric disorders, including depression (18.5–61.0%),^152^ obsessive-compulsive disorder (1.6–18%), psychotic disorders (0.1–9.8%),^153^ and substance-use disorders (34.9–51.0%),^154,155^ in TBI of all injury severities.

Among the youth population, those with TBI show increased risk of comorbidity as well, particularly with ADHD and mental health issues.^156^ Children in particular are at increased risk for psychological consequences from TBI,^157^ including social/behavioral disorders (odds ratios ranging from 1.40 to 27.11 at 12-month follow-up),^158^ increased stress responses (e.g., PTSD or other anxiety disorders), and other behavioral problems.^42^ Most mTBI studies in children have reported these effects within a few years of injury. Although a birth cohort study has offered insight into the occurrence of health and social outcomes of TBI during childhood, it is unclear how this applies to children who experience mTBI.

Importantly, psychiatric comorbidities can affect TBI recovery and the effects of TBI over time. Persons who experience a TBI and associated comorbidities show symptom overlap, and variability in symptom presentation, making it challenging to address the chronic symptoms of a TBI. Unfortunately, despite indications that TBI increases the risk of psychiatric disorder, it remains unclear and poorly understudied how mTBI contributes to the increased risk of developing comorbid psychiatric disorders in both adults and children. A recent study that examined adults with mTBI seen in an ED setting indicated that pre-injury psychiatric issues contribute to more-severe TBI symptoms and psychiatric symptoms at 3- and 6-months post-injury,^159^ emphasizing the importance of taking a medical history at the time of diagnosis to better understand psychiatric outcomes related to mTBI.

## Association of traumatic brain injury with post-traumatic stress disorder

Many TBIs occur as a result of traumatic events (e.g., interpersonal assaults, motor vehicle crashes, domestic violence, or military combat), and this can lead to a neuropsychiatric dimension of the brain injury. Epidemiological data confirm that PTSD can develop after a TBI within both the civilian and military populations. One study with >1000 patients presenting at a civilian trauma center reported that patients with an mTBI were twice as likely to develop PTSD 1 year later compared to those who did not have a TBI.^160^ Another study examined the incidence and factors associated with PTSD in participants with mTBI from the TRACK-TBI study; ~27% of patients with an mTBI screened positive for PTSD at 6 months post-injury.^53^ Screening positive for PTSD was significantly associated with concurrent functional disability, post-injury psychiatric symptomatology, decreased satisfaction with life, and decreased performance in visual processing and mental flexibility.

Among military personnel with a history of TBI of any severity, 32–39% meet the diagnostic criteria for PTSD.^161^ Indeed, in a sample of nearly 50,000 U.S. Air Force members, those who presented with mTBI (n = 5065; 10.2%) were at increased risk of PTSD.^162,163^ PTSD symptoms typically emerge 1–3 months after TBI and peak at ~6 months.^53^ With the strong association between these disorders, their symptom overlap, and their combined negative effect on outcomes, it will be important to better understand the relationship between TBI and PTSD. Existing research has applied functional neuroimaging to distinguish PTSD and TBI patients from healthy controls, identify separate signatures for TBI and PTSD, and detect their co-occurrence in highly comorbid samples^112,143,164^ (Wilde and colleagues, this issue). Thus, neuroimaging approaches may be useful for identifying and understanding the connections, overlap, and distinct comorbidity patterns in persons with TBI.

## Conclusion

TBI is common and has a large individual and societal burden because of its high prevalence, risk for long-term effects of the injury, loss to the work force, burden to healthcare systems, and impact on enviromnetal issues such as family burden, social participation, and health inequities. Comorbidities abound, affect outcomes, and confound efforts to define outcomes that are uniquely associated with TBI. Most studies examining TBI outcomes have focused on trajectories for persons experiencing moderate-to-severe TBI at the time of injury; however, research on the longer-term consequences of mTBI is emerging across the life span and is a critical component to understanding the overall burden of TBI. Despite advances in our understanding of mTBI, including epidemiological factors contributing to disease progression, new therapies to treat the consequences of the long-term sequelae are limited. The factors associated with TBI outcomes are complex and include characteristics not only of the injury, but also of the patient, such as age, pre-injury status, sex, comorbid conditions, environment, and propensity, for resilience to support recovery. Moreover, there is limited research on adult outcomes for persons who experienced a TBI during childhood, a time of rapidly changing development. Currently available reports indicate lower levels of subsequent employment and education as well as a high rate of involvement with the justice system for adults reporting a childhood TBI. Perhaps having a clear understanding of these factors will assist future clinical research designs and allow for more personalized testing of therapeutics to treat the long-term sequelae of mTBI.

Several future research directions could advance our understanding of the epidemiology of TBI.^165^ For example, we lack a complete understanding of the nexus of TBI and resilience; the influence of race, ethnicity, and sex on TBI outcomes; and the interaction of genetics, biomarkers, and behavior over time. Longitudinal global assessments on incidence, recovery, and treatment would also propel the research forward. Continued long-term outcome studies should expand the work to examine mTBI in the general population across the life span. Another important need is the development of standardized, validated assessment and outcome metrics, as well as a central organization to collect and interpret results of ongoing efforts.

Finally, future work should explore the biological underpinnings of the injury and trajectories for symptom improvement while considering individual behavior, medical history, and environment. Existing taxonomies focus on severity as mild, moderate, or severe based on duration of LOC and post-traumatic amnesia and measures such as the GCS or Abbreviated Injury Scale.^166–168^ Although each approach has demonstrated strengths, they also have weaknesses.^169^ For instance, an analysis of the CENTER-TBI European cohort study^170^ used a clustering approach with injury information and physiological data from the first 2 weeks after injury and found four severity-focused clusters that were informed not only by the GCS, but also by the mechanism of injury and presence of extracranial injury. Thus, more work is needed to use the diverse existing data available to develop a taxonomy of TBI that incorporates injury mechanisms and early biomarkers to develop standard phenotypes and improve symptom trajectories (see Pugh et al. and Wilde et al., same issue). For clinical relevance, we summarize our findings with eight takeaways and, as part of our roadmap, four trackable opportunities for next steps that include specific action items (Table 1).

- TBI poses a large burden because of high prevalence, risk for long-term effects of the injury, loss to the work force, burden to healthcare systems, and increased impact on issues such as family burden, social participation, and health inequities. mTBI represents the highest number of cases, with significant morbidity.
- Most studies examining TBI outcomes have focused on trajectories for persons experiencing moderate-to-severe TBI at the time of injury; however, research on the longer-term consequences of mTBI is emerging across the life span and is a critical area for focused research.
- Research examining mTBI includes studies that examine sports-related and non-sports-related injuries. Although caused by different injury mechanisms, it is important to consolidate these findings as related to mTBI.
- In an effort to facilitate follow-up and management, the field has described TBI as a chronic health condition based on research on moderate-to-severe cases. Research on the prolonged and longer-term health effects related to mTBI is emerging and may offer insights on the importance of follow-up and optimal management over time even for less-severe injuries.
- Examining the vestibular and oculomotor effects of mTBI is an emerging area of clinical assessment and research that may contribute to predicting long-term outcomes.
- Across the life span, children and adults can experience multiple mTBIs that can compound the long-term effects of mTBI.
- The factors associated with all TBI outcomes are complex and include not only characteristics of the injury, but also age, pre-injury status, and comorbid conditions.
- Comorbidities abound, affect outcomes, and confound efforts to define outcomes that are uniquely associated with TBI.

## Figures and Tables

**Table 1.** Actionable Research Opportunities for Epidemiological Studies in TBI

Roadmap: Opportunities for next steps	Action
Consider how injury severity, mechanism of injury, age, and lifetime TBI history could influence chronic outcomes.	Develop taxonomy of TBI based on biological impact, disease mechanism, and/or longitudinal course, rather than on injury severity. Consider how population characteristics affect the etiology of the course and outcome of TBI.
Consider how epidemiological factors contribute to patient diagnosis and heterogeneity.	Identify putative areas of interventional investigation around the presence of comorbid conditions, which suggests the potential for a shared underlying biology. Consider the opportunities and challenges of patients who present with multiple health conditions in future clinical trials’ inclusion and exclusion criteria. Develop longitudinal studies in diverse populations (e.g., pediatric, military, sports, and elderly) and identify outcome trajectories and differences in the mechanisms of injury.
Leverage new approaches for data collection.	Identify new approaches for data collection in clinical care settings, including the type of care received, use of wearable devices, and methods (e.g., machine learning, artificial intelligence) to analyze the complex data and understand persons at risk for phenotypes representing poor outcomes.
Build a national TBI registry to enable monitoring of trends in health, resources, allocation, and priority setting; ensure better data collection; and establish best practices for intervention timeliness, monitoring, and evaluation.	Develop a database or registry (similar to TBI Model Systems) that follows persons over time. Ensure that the registry follows all severities of TBI, how patients were enrolled, and tools to track those with mild injuries or those who did not receive rehabilitation services.

TBI, traumatic brain injury.
